# Manual Massage Therapy for Patients with COPD: A Scoping Review

**DOI:** 10.3390/medicina55050151

**Published:** 2019-05-17

**Authors:** Massimiliano Polastri, Enrico Maria Clini, Stefano Nava, Nicolino Ambrosino

**Affiliations:** 1 Medical Department of Continuity of Care and Disability, Physical Medicine and Rehabilitation, St. Orsola University Hospital, 40138 Bologna, Italy; 2 Department of Medical and Surgical Sciences, University of Modena and Reggio Emilia, 41121 Modena, Italy; enrico.clini@unimore.it; 3 Department of Specialistic, Diagnostic and Experimental Medicine (DIMES), University of Bologna, 40138 Bologna, Italy; stefano.nava@aosp.bo.it; 4 Istituti Clinici Scientifici Maugeri, IRCCS, Pulmonary Rehabilitation Institute of Montescano, 27040 Montescano, Italy; nico.ambrosino@gmail.com

**Keywords:** COPD, manual massage, physiotherapy, pulmonary rehabilitation, respiratory muscles

## Abstract

*Background and objectives:* Manual massage therapy is a therapeutic option for the treatment of several pathological conditions affecting the musculoskeletal system. It has been pointed out that massage might be beneficial for chronic obstructive pulmonary disease (COPD) patients thanks to therapeutic effects primarily related to hyperemia (increased skin temperature and blood flow), and activation of the lymphatic system. The present study reports current evidence on the systemic effects of manual massage in patients with COPD. *Materials and Methods:* A scoping review was conducted on five major databases. The search went through all databases since their inception until December 2018. *Results:* Seventy-eight citations were retrieved; after the selection process was completed, seven articles were considered eligible. In patients receiving manual massage, improvements were observed in Forced Expiratory Volume in 1 s, dyspnea perception, and in the 6-min walking test. *Conclusions:* To date, the use of manual massage in patients with COPD is not supported by substantial evidence in the literature: indeed, it is proposed as a therapeutic option in association with other interventions such as physical exercise.

## 1. Introduction

Manual massage includes different techniques directed to the soft tissues such as gliding, kneading, percussion, friction, and soft tissue mobilization, and treatment duration usually ranges from 5 to 30 min [1]. Manual massage therapy is a therapeutic option for the treatment of several pathological conditions affecting the musculoskeletal system [2,3,4,5,6], and it is also used in highly specialized settings [7]. Persistent respiratory symptoms and airflow limitation characterize chronic obstructive pulmonary disease (COPD) [8]. Skeletal muscle wasting, sarcopenia, and cachexia are common in patients with COPD [9] who are prone to physical activity restrictions and deconditioning due to musculoskeletal symptoms, dyspnea, and fatigue. Exercise training is a consistent element of the treatment of patients with stable COPD in addition to being part of the best practice guidelines for optimal management [8]. It has been pointed out that massage could be beneficial for COPD patients considering its therapeutic effects primarily related to hyperemia (increased skin temperature and blood flow), and activation of the lymphatic system [10]; manual massage execution includes vibratory techniques. Manual massage is an integral part of physiotherapeutic intervention and it can easily be integrated with exercise.

Although physiotherapy for patients with COPD is largely discussed in the literature, to date, there is a scarcity of data regarding the use of manual massage—and its related effects—in this class of patients. Two previously published systematic reviews found that manual massage is a suitable therapeutic option for patients with COPD [9,11]. A recent systematic review investigating the effectiveness of manual therapy interventions (alone or added to exercise) on lung function, exercise capacity, and quality of life in COPD patients, compared to exercise programs and/or sham therapies, showed that out of 555 articles screened, six fulfilled the inclusion criteria. The study designs were heterogeneous (with different interventions), and there was a high risk of bias. No effect on lung function was found, while results on exercise capacity were contrasting. Manual therapy did not affect quality of life, although valid measures were available only in one study. Only mild adverse events were reported [12].

Although manual massage has been already partially investigated, with the present review, we additionally explored the literature to gather updated evidence on the matter. Other studies discussing manual massage were focused on manual treatment techniques, instead our research explored specifically the use of manual massage in COPD patients.

### Case Vignette

A 69-year-old male former smoker (40 pack-years) with a history of COPD and recurrent disease exacerbations (2–3 per year) came to our attention on the occasion of one of these episodes. He presented with debilitating chronic cough, worsening dyspnea, and diaphragm weakness. Forced expiratory volume in 1 s (FEV_1_) was 35% (predicted); he also showed the elevation of the shoulders together with an inspiratory posture characterized by hypertrophic accessory inspiratory muscles (scalene and sternocleidomastoid). Considering the patient’s posture, manual massage could represent a therapeutic intervention to counteract muscle weakness as typically occurs in subjects with musculoskeletal disorders. We reviewed the literature to find evidence supporting the use of manual massage in patients with COPD.

## 2. Materials and Methods

### 2.1. Search Strategy

A scoping review [13] was conducted to identify primary research studies on PubMed, Scopus, Web of Science, Cochrane Library, and Cinahl. The search went through all databases since their inception until December 2018. The present review was conducted following the 2009 preferred reporting items for systematic reviews and meta-analysis (PRISMA) statement [14]. In each database we searched for the following two Medical Subject Headings (MeSH): “COPD” and “massage”. The keywords were matched as following in a search string “massage and COPD” and were searched in the title and abstract fields in each database.

For the current review, the manual massage practice was intended as described in the National Center for Biotechnology Information’s (NCBI) definition: “the systematic and methodical manipulations of body tissues best performed with the hands for the purpose of affecting the nervous and muscular systems and the general circulation” [15]. To this end, it should be highlighted that our study was centered only on manual massage excluding any other form of manual treatment. Restrictions were applied to the documents’ language: only those articles written in English, French, Italian, and Spanish were extracted. The search was limited to studies on humans, with no restriction on gender.

### 2.2. Study Selection

Both observational and experimental studies as well as research letters and short communications were considered eligible if they met the following inclusion criteria: (i) described the use of manual massage; (ii) adult patients over 18 years of age; and (iii) patients with COPD.

Citations retrieved were excluded if: they (i) were abstracts, conference proceedings, editorials, letters to editors; (ii) did not describe the manual massage practice; and (iii) referred to manual techniques other than manual massage (i.e., reflexology, acupuncture, or manual therapy) or did not fall into the definition we used to identify the treatment [15].

### 2.3. Data Extraction

Those screened articles that passed the initial selection process and that were considered suitable as they met the inclusion criteria—after the title and abstract reading—were subjected to a full-text analysis. From each study were extracted: the first author’s name; country; year of publication; study design; the number of patients; gender of patients; mean age of patients; intervention (manual massage); pulmonary functions tests values; dyspnea score; and the distance walked during the 6-min walking test (6MWT). Two reviewers extracted the data and disagreement was resolved with consensus.

## 3. Results

### 3.1. Characteristics of the Included Studies

**Figure 1:**
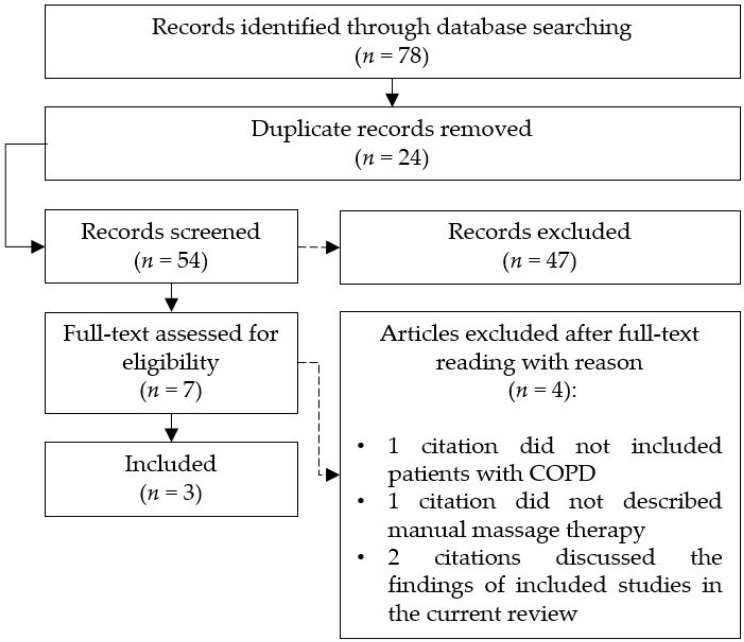
Search flow.

A total of 78 citations were retrieved by searching five databases; after the selection process was completed, a total of seven articles were screened for eligibility (Figure 1). Four articles were excluded after full-text analysis. In one case, a review investigated complementary and alternative medicine modalities, but patients with COPD were not described [16]. In another one, massage therapy was not included among treatments [17]. Eventually, the third and the fourth articles [9,11] were excluded because they discussed the findings of studies already included in the current review, and thus overlapped our research.

After the final assessment, three citations were selected for the current review: characteristics of the included studies are given in Table 1 and Table 2.

### 3.2. Manual Massage Effects

The effects of manual massage were investigated in 42 patients with COPD, the main outcome measures used in the included studies aimed at examining both respiratory and motor functions.

In the study by Szafraniec et al. [18], the manual massage was performed via percussions of the chest, and it was combined with additional therapeutic interventions (Table 2). The whole treatment was effective at improving significantly the distance walked during the 6MWT; furthermore, at the end of the therapeutic program patients reported an improved perception of their health status (Table 3). In addition, the authors found a decrease in both systolic and diastolic blood pressure (6 mmHg and 5 mmHg, respectively) after 3-weeks’ rehabilitation.

The study by Kurzaj et al. [19] described a therapeutic program lasting 6 days and including 30-min daily massage sessions. After treatment was completed, in the intervention group FEV_1_, dyspnea perception, and the distance walked improved significantly (Table 3).

In the study by Beeken et al. [20] (1998), the massage therapy was carried out along a program lasting 24 weeks. The major improvements were observed in the dyspnea intensity; the participants also reported a self-perceived better physical performance and improved relaxation, which were not supported by data. This is the only study included in which the manual massage was proposed as a stand-alone treatment as it was not associated with any other form of therapeutic intervention.

## 4. Discussion

Chronic obstructive pulmonary disease is a common, preventable, and treatable disease that is characterized by persistent respiratory symptoms and airflow limitation [8]. Typically, patients with COPD present prominent upper girdle musculature with increased work during inhalation. Moreover, the exhalation phase is mainly sustained by the abdominal muscles’ function to counteract hyperinflation and diaphragm flattening [21].

Main effects of the manual massage have been classified into reflexive, mechanical, and psychological, resulting in vasodilation and related improved circulation, decrease in pain through the release of endogenous opiates, decreasing muscle tightness, enhancing secretion clearance, and general well-being. At the same time, manual massage does not produce effects on muscle strength [1].

Chronic obstructive pulmonary disease patients are typically prone to muscular dysfunction (upper girdle, diaphragm); secretions can also be present. Manual massage effects on soft tissues are usually related to muscle relaxation which can be obtained with gliding, kneading, and friction maneuvers. At the same time, percussions and vibrations on the chest can enhance mucus clearance.

From the present study, it emerged that manual massage were primarily used as an ancillary treatment in association with other physical and pharmacological interventions. Improvements were obtained in FEV_1_, dyspnea perception, and distance walked in the 6MWT. Nevertheless, our findings should be considered with caution because only 42 patients underwent massage therapy, and this is a small sample to draw reliable conclusions. It is well known that physical training and exercise implementation are effective to ameliorating physical performance and to promote physical activity in COPD patients [22,23,24,25,26]. Although manual massage produces muscle relaxation and local hyperemia [10], its role in patients with COPD is not currently supported by substantial evidence, due to the scarcity of data.

Nevertheless, considering the status of the upper girdle musculature that characterizes patients with COPD, massage can be effective at reducing the tension of specific muscles such as trapezius, pectoralis, and sternocleidomastoideus. In this regard, the study by Kurzaj et al. [19] (2013) seems to be one of the most interesting because the intervention was well described providing detailed information about duration, techniques, and the muscle groups that were subjected to treatment. Indeed, in that study, those patients who were allocated in the intervention group obtained significant improvements both in respiratory and physical function, as shown in Table 3.

Regarding dyspnea perception, it improved in two out of the three studies included; in the research by Szafraniec et al. [18] (2016), the intensity of the shortening of breath symptom was not calculated after treatment. The distance walked increased at the 6MWT in all the studies where this measure was taken at baseline and after treatment (Table 3).

In a previously published review, Heneghan et al. [11] (2012) investigated the effects of manual treatments including manipulative techniques, massage, stretching, and passive movements; the authors concluded that additional research to provide more evidence on the use of manual therapy for COPD was needed. In the review by Heneghan et al. [11], among the analyzed studies was included that of Beeken et al. [20], which was also considered in our current research.

After the publication of the Heneghan’s [11] study, another review found that the most represented physiotherapeutic techniques used for COPD patients during hospitalization were physical and respiratory exercises including manual massage [9]; in that review, it was also included the study by Kurzaj et al. [19], which was also present in our analysis. Accordingly, to these two previously published reviews [9,11], even in our study, we did not find strong evidence supporting the use of manual massage for the treatment of patients with COPD. From a certain point of view, with the current research we are not able to add new findings to the existing literature; nevertheless, we have clarified the use of manual massage in COPD patients and our analysis can be useful to conduct further experimental research on the topic. From the present review, only in one study [20] of manual massage was used as a specific intervention, but the sample was small (5 patients), and the study was conducted before the 2000s. Indeed, the use of manual massage as a unique treatment is not feasible in COPD patients, as it is firmly accepted the concept that this population benefits from a comprehensive pulmonary rehabilitation therapeutic approach that includes several interventions [27]. One of the main problems of most of the studies performed in COPD is that the disease, or better the syndrome, may have different phenotypes and genotypes, and therefore, the effects of a technique may vary according to the subset of patients considered. Indeed, it was not clear from the literature search whether patients with different severity may respond differently to manual massage.

### Strengths and Limitations

In the current study, we were able to discuss only those findings strictly related to manual massage, which was our primary aim. If, on the one hand, our assumptions have been already partially addressed, on the other, this is the first study exploring the use of manual massage for patients with COPD specifically. Through a well-designed search strategy, we were able to optimize the literature exploration narrowing the research field.

A limitation of the present analysis can be found in the limited number of citations included in the final analysis; this was primarily related to the scarcity of data in the literature.

## 5. Conclusions

To date, the use of the manual massage in patients with COPD is not supported by substantial evidence in the literature: indeed, it is proposed as a therapeutic option in association with other interventions such as physical exercise. Although massage is one of the most known manual techniques, its use in patients with COPD should be further investigated to demonstrate its effectiveness, particularly when referring to the respiratory function.

## Figures and Tables

**Table 1:** Included studies and demographic characteristics of patients with COPD at baseline.

Variables	Authors, year [Reference], (Country)		
	Szafraniec et al., 2016 [18], (Poland)	Kurzaj et al., 2013 [19], (Poland)	Beeken et al., 1998 [20], (USA)
Study design	Interventional (pre-post study)	RCT	Case series
Number of participants	17	20	5
Gender (*n* of F)	(10)	(9)	(1)
Age (years), (mean ± SD), <min–max>	(52 ± 3)	(57 ± 7)	<57–74>
GOLD classification (stage)	(I, II)	(II, III)	-
Dyspnea intensity (MBS, mean ± SD), [MRC, mean ± SD], <VAS, min–max>	(2.4 ± 2.1)	[2.10 ± 0.77]	<0–5>
FEV_1_ (% predicted) (mean ± SD)	-	(45.2 ± 19.04)	(44.4 ± 10.9)
FVC (%)	-	-	(65.4 ± 13.0)
6MWT (meters) (mean ± SD)	(479.4 ± 17.84)	(241.0 ± 78.8)	-
Perception of health at baseline (VAS) (mean)	(7.3)	-	-

COPD: chronic obstructive pulmonary disease; Ref: reference; RCT: randomized controlled trial; F: female; SD: standard deviation; GOLD: Global Initiative for Chronic Obstructive Lung Disease; MBS: Modified Borg Scale; MRC: Medical Research Council; VAS: visual analogic scale; FEV_1_: forced expiratory volume in 1 s; FVC: forced vital capacity; 6MWT: six-minute walking test.

**Table 2:** Modalities of manual massage technique reported in the literature.

Authors, year [Reference]	Intervention
Szafraniec et al., 2016 [18]	Three-week program including chest massage (provided each day during the stay). In addition, patients received water inhalations (0.32% bicarbonate–sodium), Sollux lamp irradiation of the chest, aerosol therapy by bronchodilators (solution of 0.5 mL Berodual/3 mL 0.9% NaCl), magnetotherapy, breathing exercises, and 15-min walk.
Kurzaj et al., 2013 [19]	Six sessions of manual massage (30 min each) for the following muscles: sternocleidomastoideus, pectoralis (major and minor), trapezius, levator scapulae, rhomboids, serratus anterior, and intercostales externi. Patients were also subjected to chest relaxation exercises, abdominal exercises combined with prolonged exhalation, active exercises of peripheral joints, and walking.
Beeken et al., 1998 [20]	Neuromuscular release massage therapy: application of pressure and resistance applied to muscle groups. Gentle pressure applied to trigger points, diaphragmatic release through pressure application to the diaphragm during exhalation. Treatment was carried out by a certified massage therapist and lasted 24 consecutive weeks: therapeutic sessions were provided once a week for 1 h each.

**Table 3:** Outcomes measures following manual massage technique as reported in the literature.

Variables (After Treatment)	Authors, Year [Reference], (Country)		
	Szafraniec et al., 2016 [18], (Poland)	Kurzaj et al., 2013 [19], (Poland)	Beeken et al., 1998 [20], (USA)
Dyspnea [MRC, mean ± SD] <VAS, min–max>	-	[1.20 ± 0.83]	<0–10>
∆	-	1.10	5
FEV_1_ (%) (mean ± SD)	-	(59.2 ± 14.18)	(42.2 ± 12.7)
∆	-	14	2.2
FVC (%)	-	-	(65.8 ± 13.9)
∆	-	-	0.4
6MWT (meters) (mean ± SD)	(488.8 ± 17.63)	(318.7 ± 73.6)	-
∆	9.4	77.7	-
Perception of health (VAS) (mean)	(7.3)	-	-
∆	1	-	-

MRC: Medical Research Council; SD: standard deviation; VAS: visual analogic scale; ∆: delta versus baseline; FEV_1_: forced expiratory capacity in 1 s; FVC: forced vital capacity; 6MWT: 6-min walking test.

## References

[1] Strax T.E., Grabois M., Gonzales P., Escaldi S.V., Reyna M., Cuccurullo S.J., Cuccurullo S.J. (2014). Physical modalities, therapeutic exercise, extended bed rest, and aging effects. Physical Medicine and Rehabilitation Board Review.

[2] Barreto D.M., Batista M.V.A. (2017). Swedish massage: A systematic review of its physical and psychological benefits. Adv. Mind. Body. Med..

[3] Imai N., Ito T., Suda K., Miyasaka D., Endo N. (2017). Manual calf massage and passive ankle motion reduce the incidence of deep vein thromboembolism after total hip arthroplasty. J. Orthop. Sci..

[4] Molouki A., Hosseini S.M., Rustaee M., Tabatabaee S.M. (2016). The immediate effects of manual massage of forearm on power-grip strength and endurance in healthy young men. J. Chiropr. Med..

[5] Weerapong P., Hume P.A., Kolt G.S. (2005). The mechanisms of massage and effects on performance, muscle recovery and injury prevention. Sports. Med..

[6] Nordschow M., Bierman W. (1962). The influence of manual massage on muscle relaxation: Effect on trunk flexion. J. Am. Phys. Ther. Assoc..

[7] Polastri M., Savini C., Grigioni F. (2013). Calf cramps in a heart transplant patient during the postoperative course: A case report. IJTR.

[8] Global Initiative for Chronic Obstructive Lung Disease. https://goldcopd.org/wp-content/uploads/2018/11/GOLD-2019-v1.6-FINAL-08Nov2018-wms.pdf.

[9] De Alvarenga G.M., Remigio Gamba H., Elisa Hellman L., Ganzert Ferrari V., Michel de Macedo R. (2016). Physiotherapy intervention during level I of pulmonary rehabilitation on chronic obstructive pulmonary disease: A systematic review. Open. Respir. Med. J..

[10] Olszewska J. (2011). Rehabilitation for chronic obstructive pulmonary disease patients. Pol. Ann. Med..

[11] Heneghan N.R., Adab P., Balanos G.M., Jordan R.E. (2012). Manual therapy for chronic obstructive airways disease: A systematic review of current evidence. Man. Ther..

[12] Simonelli C., Vitacca M., Vignoni M., Ambrosino M., Paneroni M. (2019). Effectiveness of manual therapy in COPD: A systematic review of randomized trials. Pulmonology.

[13] Munn Z., Peters M.D.J., Stern C., Tufanaru C., McArthur A., Aromataris E. (2018). Systematic review or scoping review? Guidance for authors when choosing between a systematic or scoping review approach. BMC Med. Res. Methodol..

[14] Moher D., Liberati A., Tetzlaff J., Altman D.G., PRISMA Group (2009). Preferred reporting items for systematic reviews and meta-analyses: The PRISMA statement. PLoS Med..

[15] National Center for Biotechnology Information Massage. https://www.ncbi.nlm.nih.gov/mesh/68008405.

[16] Pan C.X., Morrison R.S., Ness J., Fugh-Berman A., Leipzig R.M. (2000). Complementary and alternative medicine in the management of pain, dyspnea, and nausea and vomiting near the end of life. A systematic review. J. Pain. Symptom. Manag..

[17] Bailey C.D., Wagland R., Dabbour R., Caress A., Smith J., Molassiotis A. (2010). An integrative review of systematic reviews related to the management of breathlessness in respiratory illnesses. BMC Pulm. Med..

[18] Szafraniec R., Józefowski P., Chojnowska A. (2016). Ther effect of 3-week sanatorium rehabilitation on exercise capacity and subjective perception of health of patients with asthma and COPD. Balt. J. Health. Phys. Act..

[19] Kurzaj M., Wierzejski W., Dor A., Stawska J., Rożek K. (2013). The impact of specialized physiotherapy methods on BODE index in COPD patients during hospitalization. Adv. Clin. Exp. Med..

[20] Beeken J.E., Parks D., Cory J., Montopoli G. (1998). The effectiveness of neuromuscular release massage therapy in five individuals with chronic obstructive lung disease. Clin. Nurs. Res..

[21] Bellamy D., Booker R. (2011). Chronic Obstructive Pulmonary Disease in Primary Care.

[22] Demeyer H., Donaire-Gonzales D., Gimeno-Santos E., Ramon M.A., DE Battle J., Benet M., Serra I., Guerra S., Farrero E., Rodriguez E. (2018). Physical activity is associated with attenuated disease progression in COPD. Med. Sci. Sports Exerc..

[23] Rochester C.L., Vogiatzis I., Powell P., Masefield S., Spruit M.A. (2018). Patients’ perspective on pulmonary rehabilitation: Experiences of European and American individuals with chronic respiratory diseases. ERJ Open. Res..

[24] Felcar J.M., Probst V.S., de Carvalho D.R., Merli M.F., Mesquita R., VIdotto L.S., Ribeiro L.R.G., Pitta F. (2018). Effects of exercise training in water and on land in patients with COPD: A randomised clinical trial. Physiotherapy.

[25] Özmen I., Yildirim E., Öztürk M., Ocakli B., Yildiz R., Aydin R., Karakiş M., Yilmaz Ö., Aksoy E. (2018). Pulmonary rehabilitation reduces emergency admission and hospitalization rates of patients with chronic respiratory diseases. Turk. Thorac. J..

[26] Ubolsakka-Jones C., Pongpanit K., Boonsawat W., Jones D.A. (2019). Positive expiratory pressure breathing speeds recovery of postexercise dyspnea in chronic obstructive pulmonary disease. Physiother. Res. Int..

[27] Spruit M.A., Singh S.J., Garvey C., ZuWallack R., Nici L., Rochester C., Hill K., Holland A.E., Lareau S.C., Man W.D. (2013). An official American Thoracic Society/European Respiratory Society statement: Key concepts and advances in pulmonary rehabilitation. Am. J. Respir. Crit. Care. Med..

